# Integrated TOPSIS-COV approach for selecting a sustainable PET waste management technology: A case study in Qatar

**DOI:** 10.1016/j.heliyon.2022.e10274

**Published:** 2022-08-23

**Authors:** Nayla Ahmad Al-Thani, Tareq Al-Ansari, Mohamed Haouari

**Affiliations:** a Division of Sustainable Development, College of Science and Engineering, Hamad Bin Khalifa University, Qatar Foundation, Doha, Qatar; b Mechanical and Industrial Engineering Department, College of Engineering, Qatar University, Doha, Qatar

**Keywords:** Performance indicators, Sustainable waste management, Analytic hierarchy process, Multi-criteria decision making, Resource management, Circular economy

## Abstract

In 2018, the global annual consumption of Polyethylene terephthalate (PET) bottles was approximately 27.64 million tons, with one million bottles sold worldwide every minute. Unmanaged PET bottles in the environment lead to a series of negative effects on the health of humans and ecosystems. Therefore, the objective of this research was to evaluate the sustainability of eight different PET waste bottle treatment methods using a holistic multi-criteria decision-making approach that combined the technique for order of preference by similarity to ideal solution (TOPSIS) with analytic hierarchy (AHP; TOPSIS-AHP) and coefficient of variation (COV; TOPSIS-COV) approaches. To the best of our knowledge, TOPSIS-COV has not yet been used for waste management. The treatment methods were compared and analyzed against twelve different performance criteria representing three pillars of sustainability: environmental, economic, and social. Both approaches determined closed-loop recycling to be optimal for treating PET waste bottles. The weights of performance indicators obtained using the COV and AHP approaches were comparable, except for cost, photochemical oxidant potential, and human toxicity. The large dispersion in the values of the photochemical oxidant potential causes it to have a higher weight in the COV approach. For cost, the weight was higher using the AHP approach by approximately 12%, which reflects the preference of decision-makers to reduce costs of ventures.

## 1 Introduction

Since 1974, when the first polyethylene terephthalate (PET) bottle was produced (Parker, 2019), the demand for PET bottles has drastically grown. Every minute, one million plastic bottles are sold worldwide (de Marchi et al., 2020). The PET bottled water market is forecasted to become the largest by volume worldwide in the bottled drink category (de Marchi et al., 2020). In 2018, the global annual consumption of PET bottles was approximately 27.64 million tons (Wang et al., 2020). The total production of plastic over the last 65 years is approximately 8300 million metric tons (Geyer et al., 2017), and approximately 79% of this currently exists in landfills (de Marchi et al., 2020). The rapid increase in the demand for PET bottled water can be linked to different factors, such as the growing scarcity of drinkable water, increased standards of living, and the unique properties of PET bottles (Lendvai et al., 2022). PET bottles have several properties that make them suitable for packaging water and other beverages; the properties include their alkali and acid resistance, high strength with light weight, and being inexpensive to produce (Wang et al., 2020). In addition, PET has good transparency and dimensional stability, is a good barrier against moisture and oxygen, and is nontoxic (Majumdar et al., 2020). However, the rapid production and consumption of PET bottles have resulted in disastrous consequences for the environment and human health owing to the large amount of waste abandoned in ecosystems. The amount of waste generated by a country positively correlates with its income, and the share of plastic waste in this total waste also increases with income (World Bank Group, 2018).

Unmanaged PET bottles abandoned in the environment lead to negative effects on human health and ecosystems (i.e., air, soil, and water). In recent years, the effects of plastics and other chemicals added during the production process on human health have been studied (Pjanic, 2017). PET bottles have been linked with insulin resistance (Pjanic, 2017), decreased anogenital distance in male infants (Wagner and Oehlmann, 2009), lower levels of sex hormones (Sax, 2010), and adverse effects on the immune system, particularly in young people (Rustagi et al., 2011).

The effects of discarded plastics on ecosystems are more obvious and severe. Mismanaged plastic has contaminated large bodies of fresh water, rivers, and oceans. There is approximately 6.3 billion metric tons of plastic waste debris worldwide (Geyer et al., 2017) accounting for 60–80% of total global waste (de Marchi et al., 2020). In 2012, there was approximately 165 million tons of plastic waste in the oceans. In 2050, it is estimated that there will be 5.25 trillion plastic particles in the oceans (Qatar Development Bank, 2017) with a combined weight exceeding that of fish (Alabi et al., 2019). The extended breakdown of plastics leaches toxic chemicals and microparticles into water bodies and soil. Plastic microparticles have been detected in the guts of fish in the Arabian Gulf (Al-Salem et al., 2020). Although PET bottles are non-toxic and are widely used in the food, beverage, and textile industries, their mismanagement is a major problem and needs to be addressed. The negative impact of PET waste has been growing for many reasons, such as the decreasing amount of available landfill space, ineffective methods for removing PET waste from the environment (especially oceans), and the blockage of sewage systems (Alabi et al., 2019). Furthermore, in recent years, there has been a move toward limiting the amount of waste that is landfilled due to leaching to the soil and ground water, even with good management, which can cause harmful contamination that impacts agriculture, human health, and wildlife (Alabi et al., 2019).

Waste management is at the core of sustainable development and impacts environmental, economic, and social dimensions. The increasing competition for natural resources is underpinned by the interlinkages that exist between them, and the scarcity of natural resources emphasizes the need to develop systematic waste management techniques. The management of natural resources forms the core of complex decision making related to sustainable production. There is a need to adopt systematic scientific-based approaches and frameworks to manage resources and their complexities. It is a well-documented fact that, despite its disastrous environmental impact, landfilling remains by far the most popular end-of-life process for PET in most developing countries. Apparently, and based on personal discussions with decision makers in Qatar, the main reason seems to be the general belief that landfill is the most economical end-of-life process and that any other environmentally friendly process is significantly more expensive and therefore unaffordable. Therefore, this study develops a multi-criteria decision-making (MCDM) approach for managing PET by analyzing the pathways of PET waste management at the strategic level. The aim of this approach is to identify optimal end-of-life treatment alternatives for PET waste management by evaluating them against a set of sustainability performance indicators. To achieve this, we will develop a MCDM approach to identify the optimal technology for PET waste treatment.

The remainder of this paper is organized as follows: Section 2 presents a literature review for multi-criteria decision analysis of waste systems; Section 3 describes the methodology and materials used; Section 4 presents and discusses the results of the case study; finally, Section 5 provides concluding remarks.

## 2 Multi-criteria decision analysis of waste systems

Waste Management Systems (WMS) are complex systems, due to the large number of interrelationships between the various elements of the system. As the complexity of the system increases the decision-making process becomes more challenging, since all decisions imply a different prediction and outcome of the future, and as such more systematic approach is required (Bennet and Bennet, 2008). In order to support decision-makers in planning, managing, and facilitating decisions of WMS in an optimized and structural manner, Multi-Criteria Decision Making (MCDM) approaches often are utilized. In this section, we will review the literature related to the application of MCDM approaches to waste management. We will first focus on applications related to municipal solid waste (MSW) management and then review the literature related to plastic waste management. We conclude this section by highlighting some gaps that other researchers have not yet addressed.

### 2.1 Municipal solid waste management applications

Municipal Solid Waste (MSW) gained the most attention from the researchers due to the ever-increasing amount generated of MSW worldwide. It is estimated that the amount of MSW will reach 2.2 billion tonnes in 2025 worldwide (Chen et al., 2022). Thus, many researchers studied how to optimize the MSW systems utilizing different MCDM and optimization techniques. For instance, (Aghajani Mir et al., 2016) proposed a revised TOPSIS approach to determine the optimal treatment technique for Municipal Solid Waste (MSW). The study utilized the VIKOR method (Opricovic and Tzeng, 2004) to perform a sensitivity analysis since the preference of the decision-maker was unknown. The study considered landfill, open dump, refuse derived fuel, anaerobic digestion, composting, incineration, and recycling as different possible scenarios for MSW management in Malaysia. Similarly, (Coban et al., 2018) conducted a comprehensive analysis to determine the optimal technique for MSW disposal. The study utilized three different MCDM approaches, namely TOPSIS and Preference Ranking Organization Method for Enrichment Evaluations PROMETHEE I and PROMETHEE II to ensure the reliability of the selected scenario under the three different approaches. Focusing on scenario analysis, (Estay-Ossandon et al., 2018) developed a comparative model that integrates the Delphi method, fuzzy TOPSIS, and system dynamics to assess the different possible MSW treatments. The main objective was to enhance the process of MSW planning and forecasting. Moreover, (Govind Kharat et al., 2019) utilized fuzzy methods to determine the optimal MSW treatment options for India. The methods used in the study were fuzzy Delphi Method to identify the relevant criteria and the most suitable treatment options, then fuzzy AHP to set the weights for each criterion, and finally fuzzy TOPSIS to rank the alternatives. The study considered bio-methanation, gasification-pyrolysis, landfill, composting, incineration, and refuse-derived combustion. (Shahnazari et al., 2020) combined AHP and TOPSIS to identify the optimal technique for thermochemical MSW management. The techniques considered in the research were plasma, pyrolysis, gasification, and incineration under environmental, economic, and technological criteria.

For the solid waste management, in 2017, (Xu et al., 2017) developed a robust formulation for the global reverse supply chain for recycling solid waste. The formulation included mixed-integer linear programming formulation (MILP) to minimize the total cost of handling and transportation, while maintaining the emissions under pre-defined levels. Combining linear programing and MCDA (Asefi and Lim, 2017) develop a holistic model that combines TOPSIS and multi-objective optimization. The objective of the model was to design an integrated solid waste management system by allocating the optimal facility location.

Allocation of multi-waste streams was investigated by (Yılmaz Balaman et al., 2018) In 2018. In their research they developed a fuzzy linear programming model that aims to optimize multi-waste supply chain within the circular economy framework. The developed model decides on the structure of the supply chain, the best technology for the production, and production and distribution planning for a waste to bioenergy supply chain at a regional level. The objectives considered in the model are: maximizing the profit, minimizing the total capital investment cost, and minimizing the environmental impact and GHG emissions. (Gambella et al., 2019) developed a two-stage multi-period stochastic mixed-integer programing formulation, the developed model aims to support tactical decision-making (one-year plan) for waste flow allocation from the waste operator's perspective. The objective of the model is to determine the optimal pre-allocation of the waste flow, while minimizing the total cost considering the possible profit from recycling and resource recovery, in addition to the possibility of treating the excess waste outside the network as a corrective action.

TOPSIS has been used extensively in the analysis of the other waste streams such as the health care waste, where (Manupati et al., 2021) used fuzzy TOPSIS and fuzzy VIKOR to optimally handle the health care waste. The research considered nine different techniques and ten different criteria. In the area of the food waste, (Wohner et al., 2020) used MCDM to investigate the food waste and loss in the agriculture industry while considering environmental and economic criteria. The study used TOPSIS to find the most sustainable method for the packaging. Moreover, for waste-to-energy, (Alao et al., 2020) utilized the TOPSIS method to determine the optimal technology for waste-to-energy in Nigeria. The weights of the criteria were determined using the entropy method. Later in 2021, (Alao et al., 2021) proposed a hybrid MCDM method that combines the integrated determination of criteria weights (IDOCRIW) with weighted TOPSIS to identify the optimal waste-to-energy option. The study proposed to integrate the criterion impact loss (CILOS) and the entropy method, as well to determine the weights of the criteria. The authors suggested that the combination will overcome limitations of the entropy method. (Afrane et al., 2021) performed a techno-economic analysis in order to inform decision-makers on the optimal waste-to-energy technology to be adopted in Ghana. The study utilized a fuzzy TOPSIS, which provides a range of weights instead of one number. The study considered four different technologies which are anaerobic digestion, gasification, pyrolysis, and syngas based on ten different criteria.

### 2.2 Plastic waste management applications

Plastic represents about 12% of the global generated waste in 2016 (World Bank Group, 2018). As such, in recent years plastic waste management has emerged as an urgent issue that must be addressed because of the disastrous effect it has on the environment and human health.

In this context, several authors have used multi-criteria decision-making (MCDM), as well as mixed integer programming (MIP), to optimize waste management operations. In this section, we briefly review the relevant applications of MCDM and MIP to plastic waste management (Huang et al., 2011).

There are many studies focusing on optimizing one technology such as, (Rentizelas et al., 2018) illustrated through experiments the use of pyrolysis technology and the useful outcomes from the plastic used in the agriculture. After which, a robust mixed-integer linear programming was developed to optimize the location of the pyrolysis plants, the capacity of the plants and the suppliers' allocation and customers' allocation to the plans, while maximizing the Net Present Value. Focusing on the supply chain system of the plastic waste,

(Balwada et al., 2021) implemented an AHP approach to determine the optimal plastic waste collection method to support the circular economy in India. (Geetha et al., 2021) proposed a new model called “*Hesitant Pythagorean Fuzzy - ELimination and Choice Expressing REality III*” in order to identify the optimal disposal option for different plastic polymer types. The study considered four different alternatives, which were mechanical, chemical, feedstock and incineration for energy, while the polymers considered were PET, HDPE, PVC, LDPE, PP, and PS. (Deshpande et al., 2020) utilized the Multi-Attribute Value Theory to decide on the optimal end-of-life treatment for the plastic waste resulted from the fishing industry in Norway. The EOL alternatives utilized are landfilling, incinerating, and recycling.

The combination of AHP and TOPSIS has been utilized in selecting the optimal strategy for waste management (Pires et al., 2011), as well as selecting the best alternative for waste management. (Vinodh et al., 2014) focused on recycling mixed plastic waste by integrating a Fuzzy AHP approach with TOPSIS approach to determine the optimal technique. The Fuzzy AHP approach was used to determine the weights of each criterion, whilst the TOPSIS approach identifies the best alternative based on the weights developed from AHP. The considered alternatives were chemical recycling, mechanical recycling, and energy recovery. The study identified 20 different criteria based on the feedback from the decision-makers. Table 1 below summarizes the difference between this research and the previous research.

**Table 1:** Comparison between other research and this research.

Reference	Method					Performance Criteria				Technology	Material
	AHP	Fuzzy AHP	TOPSIS	COV	Other	Environment	Economic	Social	Technical		
(Vinodh et al., 2014)		X	X			Air resources, Water resources, Land resources, Mineral and energy resources,	Economic performance, financial benefits, Trading opportunities, Macro social performance. Managerial ability, Interest support groups, Customer Satisfaction, Managerial effectiveness, Management ability	Health, Potential Internal human resources, External population, Stakeholder population,	Technical capability, New technology acceptance, technical support and training	Mechanical recycling process Chemical recycling process Energy recovery	Mixed Plastic

(Rentizelas et al., 2018)					Robust mixed- integer linear programming					Pyrolysis	

(Shahnazari et al., 2020)	X		X			Slag Land use Emissions		Employment rate Accessories industry growth Operational cost	Technology level Ease of equipment access Ease of technology use Workplace safety	Plasma Pyrolysis Gasification Incineration	Municipal Solid Waste

(Deshpande et al., 2020)					Multi-Attribute Value Theory					landfilling, incinerating, and recycling	Mixed Plastic

(Geetha et al., 2021)					Hesitant Pythagorean Fuzzy - ELimination and Choice Expressing REality III”					Mechanical, chemical, feedstock and incineration for energy	PET, HDPE, PVC, LDPE, PP, and PS

This research	X		X	X		Global warming potential, abiotic depletion potential, marine ecotoxicity potential, freshwater ecotoxicity potential, terrestrial ecotoxicity potential, eutrophication potential, terrestrial acidification potential, photochemical oxidant formation, and ozone depletion potential.	Cost Revenue	Human toxicity potential.		Closed-loop, open-loop (mechanical), open-loop (semi-mechanical), landfill, incineration with heat recovery, incineration with heat and power recovery, glycolysis, and pyrolysis.	PET bottles

From Table 1, it is clear that there is a lack of studies focused on optimizing PET waste streams, hence the need for further research in the area of PET bottle waste management. To our knowledge, there are no studies in the literature that combine all primary, secondary, tertiary and quaternary recycling alternatives by specific technologies against competing sustainability performance indicators for PET specifically. In addition, the combination of TOPSIS and the VOC method for calculating performance criteria weights has not previously been used in the waste management field.

## 3 Materials and methods

**Figure 1:**
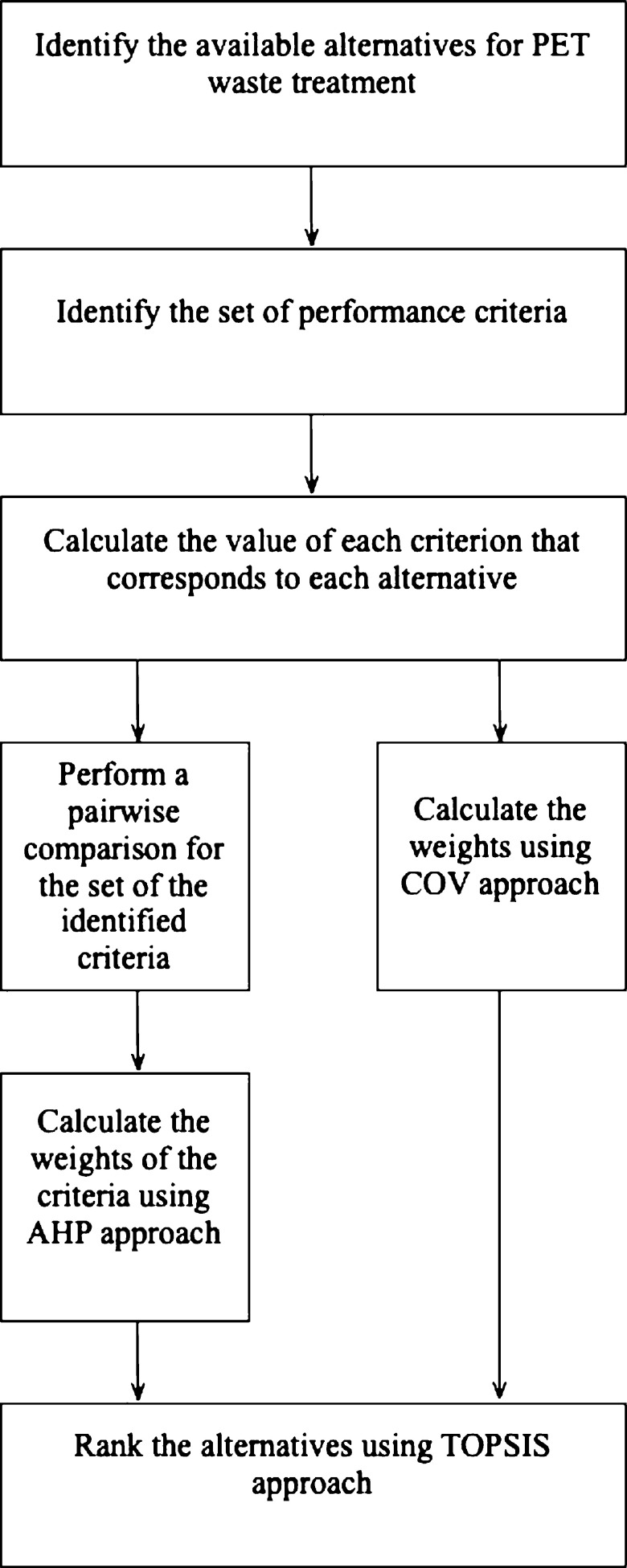
Methodological framework.

In this study, an MCDM method combining TOPSIS, AHP, and COV was used to determine the optimal end-of-life treatment options for PET waste bottles. AHP was utilized to calculate subjective weights based on the decision-maker's preference. However, COV has not been used in the waste management domain, particularly when selecting the best alternative from a set of alternatives according to predefined criteria. The proposed methodology consists of multiple steps, as illustrated in Fig. 1. The entropy method is the most popular approach utilized in the literature to compute the objective weights; however, the approach assumes that the dataset is all positive, and in the collected decision matrix, the negative in the data presents the offset of a certain process to the performance criteria.

### 3.1 Performance indicators

As the complexity of the waste management systems increases, there is an increasing need for assessment tools to help decision makers quantify the trade-offs and impacts of different actions on a system as a whole and as sub-components. Thus, different performance measures have been developed in almost all scientific fields. Measuring the changes in a system requires the use of indicators, defined as “quantitative or qualitative factor or variable that provides a simple, and reliable, means to measure achievement, to reflect the changes connected to an intervention, or to help assess the performance of a development actor” (OECD, 2013). Indicators are vital as they can present complex, dynamic knowledge or information in focused and summarized forms by synthesizing the relevant knowledge about the phenomenon or system based on a reference value; they provide an easier way to communicate targets, objectives, and achievements and track progress (Saidani et al., 2019) to aid the decision-making process.

Table 2 presents the performance indicators selected to analyze the sustainability of PET waste treatment alternatives. The performance indicators were selected based on a systematic literature review of waste management, circular economy, and industrial ecology. The selected performance indicators covered the areas of environment, human health (social), and economics. Through the literature review, it was evident that the impact categories of life cycle assessment were the most comprehensive, capturing the different environmental impacts and providing a holistic perspective on the impact of each process on the environment. However, in the analysis, social and economic performance criteria were included to measure the degree of sustainability of each proposed PET waste treatment.

**Table 2:** Selected performance indicators.

Indicator	Description	Reference
Global Warming Potential (GWP)	Potential change in the earth's temperature due to the release of greenhouse gases.	(ISO14050, 2020)
Abiotic Depletion Potential (ADP)	Potential depletion of natural resources.	(Guinée et al., 2001)
Marine Ecotoxicity Potential (METP)	Potential impact of released toxic substances on marine environments.	(Fairbrother and Hope, 2005)
Freshwater Ecotoxicity Potential (FETP)	Potential impact of released toxic substances on freshwater organisms.	(Fairbrother and Hope, 2005)
Terrestrial Ecotoxicity Potential (TETP)	Potential impact of released toxic substances on land-dependent organisms.	(Fairbrother and Hope, 2005)
Eutrophication Potential (EP)	Potential overgrowth of plankton, algae, and higher aquatic plants due to increased nutrients in the water.	(Čuček et al., 2015)
Terrestrial Acidification Potential (TAP)	Potential impact of acidifying pollutants on soil, groundwater, surface waters, and biological organisms.	(Guinée et al., 2001)
Photochemical Oxidant Potential (POFP)	Potential formation of photochemical ozone in the lower atmosphere.	(Gad, 2005)
Ozone Depletion Potential (ODP)	Potential damage to the protective stratospheric ozone layer due to the human emitted gases.	(Wuebbles, 2015)
Human Toxicity Potential (HTP)	Potential impact of emitted substances that exist in the environment on human health.	(Guinée et al., 2001)
Cost	Estimated cost of treating one ton of PET waste.	–
Revenue	Estimated revenue of treating one ton of PET waste.	–

The values of the performance indicators were calculated by converting the emission of each waste treatment to the different impact categories using CML Characterization factors Gabathuler, 2006).

### 3.2 List of PET waste treatment

**Figure 2:**
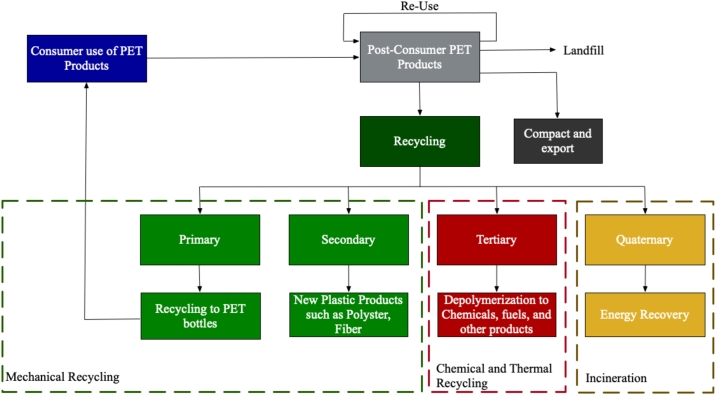
Different roots of PET waste treatment alternatives.

To identify the available technologies for PET waste treatment, an extensive literature review was conducted. All the available pathways for PET waste are illustrated in Fig. 2. PET waste treatment technologies were selected based on different criteria: the availability of process emissions in the literature, technology readiness, and suitability for treating PET waste. The alternatives considered in this research are listed in Table 3. All processes start as post-consumer PET waste bottles that need to be collected and transported to a material separation facility. Subsequently, the separated PET waste bottles underwent pre-treatment. The pre-treatment steps often included sorting, washing, drying, and shredding.

**Table 3:** Description of the selected technologies.

Technology	Description
Closed-loop recycling	Converting PET waste bottles through physical processes into new bottles with similar properties to the original product.
Open-loop recycling (Mechanical recycling)	Converting PET waste bottles using physical and chemical processes into a lower grade plastic product. In mechanical recycling fibers are produced through direct extrusion from flakes.
Open-loop recycling (semi-mechanical recycling)	Converting PET waste bottles using physical and chemical processes into a lower grade plastic product. In the semi-mechanical recycling, the first step is to utilize extrusion to make pellets, then the pellets are converted into fibers and other products.
Glycolysis	Chemical recycling technique that converts PET waste bottles into oligomer in the presence of a catalyst. Then the oligomers are repolymerized into PET fiber.
Pyrolysis	Converting PET waste bottles into other useful products, such as oils and syngas, through thermal decomposition in an inert atmosphere.
Incineration with heat recovery	Combustion of PET waste bottles as a fuel to releases heat. The heat is used for heating spaces (Chilton et al., 2010).
Incineration with heat and power recovery	Combustion of PET waste bottles as a fuel to releases power and heat. The power feeds the grid and the heat is used for heating spaces (Chilton et al., 2010).
Landfill	Transporting PET waste bottles into landfill sites; the first and most used waste management strategy in most countries including developed countries.

Below, we provide a detailed approach for all methods used in this study.

### 3.3 Technique for order of preference by similarity to ideal solution

MCDM is broadly used in problems where the performance of different alternatives should be ranked against multiple conflicting criteria (Deng et al., 2000). Many approaches for MCDM have been developed, one of which is TOPSIS, which was first proposed in 1981 (Yoon and Hwang, 1981).

The main concept of TOPSIS is to determine the alternative that has the shortest Euclidean distance to the ideal solution and the longest Euclidean distance from the negative-ideal solution (Deng et al., 2000). TOPSIS has multiple attributes that make it favorable, such as a) the approach is rational and clear, b) the calculations are straightforward and easy to compute, c) the approach is capable of identifying the optimal alternative among multiple alternatives in simplified mathematical representation, and d) the method incorporates objective methods in the calculations (Deng et al., 2000).

Throughout this paper, we use the following notation.

$A=\{A_{i},\;i=1,2,\ldots ,m\}$ set of PET waste treatment alternatives

$C=\left\{C_{j},\;j=1,2,\ldots ,n\right\}$ set of performance evaluation criteria

$x_{ij}$: performance of alternative *i* against performance criterion *j*

*X*: decision matrix, that is, performance matrix with dimensions m×n

$$X=\left[\begin{array}{ccc} x_{11}\; & \cdots \; & x_{1n}\\ \vdots \; & \ddots \; & \vdots \\ x_{m1}\; & \cdots \; & x_{mn} \end{array}\right]$$

$r_{ij}$: nondimensional attribute corresponding to $x_{ij}$

$w_{j}$: weight of criterion $C_{j}$; j=1,2,…,n

*V*: normalized decision matrix

$A^{+}$: ideal solution

$A^{-}$: negative-ideal solution.

The calculation steps of TOPSIS were as follows:

Step 1: build the normalized decision matrix.

$$r_{ij}=\frac{x_{ij}}{\sqrt{\sum_{i=1}^{m}x_{ij}^{2}}}$$

Step 2: build the weighted normalized decision matrix.

$$V=\left[\begin{array}{ccc} w_{1}r_{11}\; & \cdots \; & w_{n}r_{1n}\\ \vdots \; & \ddots \; & \vdots \\ w_{1}r_{m1}\; & \cdots \; & w_{n}r_{mn} \end{array}\right]=\left[\begin{array}{ccc} v_{11}\; & \cdots \; & v_{1n}\\ \vdots \; & \ddots \; & \vdots \\ v_{m1}\; & \cdots \; & v_{mn} \end{array}\right]$$

Step 3: identify the ideal solution and the negative ideal solution.

$$A^{+}=\left\{\left(\max_{i}v_{ij}|j\in J\right),\left(\min_{i}v_{ij}|j\in J^{\prime }\right)|i=1,2,\ldots ,m\right\}=\left\{v_{1}^{+},v_{2}^{+},\ldots ,v_{j}^{+},\ldots ,v_{n}^{+}\right\}$$

$$A^{-}=\left\{\left(\min_{i}v_{ij}|j\in J\right),\left(\max_{i}v_{ij}|j\in J^{\prime }\right)|i=1,2,\ldots ,m\right\}=\left\{v_{1}^{-},v_{2}^{-},\ldots ,v_{j}^{-},\ldots ,v_{n}^{-}\right\}$$

where, J={j=1,2,…,n|j associated with the benefit criteria}

$$J^{\prime }=\left\{j=1,2,\ldots ,n|j\text{ associated with the cost criteria}\right\}$$

Step 4: compute the ideal separation and the negative-ideal separation.

- The ideal separation

$$S_{i}^{+}=\sqrt{\sum_{j=1}^{n}{\left(v_{ij}-v_{j}^{+}\right)}^{2}}\;i=1,2,\ldots ,m$$

- The negative-ideal separation

$$S_{i}^{-}=\sqrt{\sum_{j=1}^{n}{\left(v_{ij}-v_{j}^{-}\right)}^{2}}\;i=1,2,\ldots ,m$$

Step 5: compute the relative closeness to the ideal solution.

$$C_{i}^{⁎}=\frac{S_{i}^{-}}{\left(S_{i}^{+}+S_{i}^{-}\right)},\;0<C_{i}^{⁎}<1,\;i=1,2,\ldots ,m$$

$$C_{i}^{⁎}=1\;\text{if }A_{i}=A^{+}$$

$$C_{i}^{⁎}=0\;\text{if }A_{i}=A^{-}$$

Step 6: rank the preference order according to the descending order of $C_{i}^{⁎}$.

### 3.4 Analytic hierarchy process

Analytic hierarchy process (AHP) is a method developed in 1984 to address complex decisions in complex environments (Saaty, 1987). The difficulty of decision making in complex systems stems from the multi-interactions between competing and non-competing elements of the system and the dynamic nature of complex systems. AHP is a holistic systematic framework that utilizes psychology and mathematics to measure the relative priorities of a set of alternatives (Pachemska et al., 2014). The main idea of the AHP approach is to formulate a complex problem into a hierarchical structure (Chauhan et al., 2020) that contains specific components: the goal (i.e., the objective of the problem), criteria (which are the second level), and alternatives (which are the last level of the hierarchy) (Russo and Camanho, 2015). The steps of AHP (Saaty, 1987) are as follows:

1- Define the problem and the desired type of information. It is crucial to consider all the required assumptions and perspectives to build a decision on solid ground.
2- Construct the decision hierarchy.
3- Build pairwise comparison matrices. “Each element in an upper level is used to compare the elements in the level immediately below with respect to it.” (Saaty, 1987). The pairwise comparison matrix is constructed to establish priorities for each criterion in the first level, and each criterion in the upper level will have one matrix for the respective sub-criteria.
4- Calculate the relative priorities of each element in all the levels.
5- Calculate the final weights for each alternative.
6- Check the consistency index.

**Figure 3:**
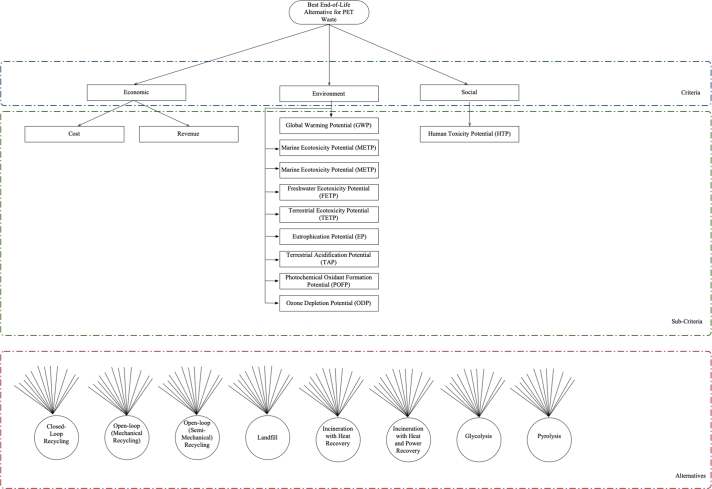
Hierarchical decision structure of PET waste management.

The hierarchical decision structure of the PET waste management system is illustrated in Fig. 3, and the corresponding performance indicators are presented in Table 4.

**Table 4:** The Performance indicators for the selected end-of-lives.

Process	Closed-Loop^1^	Open-loop (mechanical)^2^	Open-loop (semi-mechanical)^2^	Landfill^3^	Incineration with heat recovery^1^	Incineration with heat and power recovery^1^	Glycolysis^2^	Pyrolysis^5^
GWP	−1.70 × 10^3^	7.09 × 10^2^	1.31 × 10^3^	2.91 × 10^3^	4.95 × 10^3^	1.40 × 10^3^	1.84 × 10^3^	4.31 × 10^2^
ADP	−1.34 × 10^−2^	4.43	7.67	3.31 × 10^−4^	8.91 × 10^−4^	8.6 × 10^−6^	1.27 × 10^1^	2.53 × 10^−6^
METP	−1.10 × 10^4^	0.00	0.00	8.62 × 10^5^	2.34 × 10^6^	1.7 × 10^4^	0.00	4.86 × 10^3^
FETP	−7.87 × 10^1^	2.19 × 10^2^	1.74 × 10^2^	2.04 × 10^2^	5.62 × 10^2^	1.2 × 10^2^	2.15 × 10^2^	3.49 × 10^1^
TETP	−4.44 × 10^−1^	5.17	4.88	1.70	4.72	6.7 × 10^−1^	1.21 × 10^1^	1.97 × 10^−1^
EP	−1.04 × 10^−1^	5.91 × 10^−1^	4.88 × 10^−1^	3.60	4.90	−7.2 × 10^−2^	1.63	6.11 × 10^−2^
TAP	−1.84	2.22	6.28	4.34 × 10^1^	3.74 × 10^1^	−3.2	9.95	8.26 × 10^−1^
POFP	−2.09 × 10^1^	1.48 × 10^−1^	2.09 × 10^−1^	2.39	2.10	−5.9	4.26 × 10^−1^	1.16
ODP	0.00	0.00	0.00	1.18 × 10^−5^	3.16 × 10^−5^	0.00	0.00	0.00
HTP	−7.16 × 10^4^	2.68 × 10^2^	2.89 × 10^2^	1.46 × 10^2^	4.83 × 10^2^	1.1E+5	5.29 × 10^2^	3.17 × 10^4^
Cost^4^	500	500	500	1.80 × 10^2^	(4.20 × 10^2^)⁎	(2.1 × 10^2^)⁎	1300	410
Revenue	1000	1000	1000	0.00	41.4	41.36	1400	590

1 (Chilton et al., 2010).

2 (Shen et al., 2010).

3 (Aryan et al., 2019).

4 (Willard, 2019).

5 (Yoshioka et al., 2004).

⁎ (Gradus et al., 2016).

### 3.5 Coefficient of variation (COV) approach

In subjective weighting methods, the preferences and previous experiences of decision-makers influence the weights of the criteria and as the number of criteria increases, the accuracy of these subjective decision-maker preferences decreases (Keshavarz-Ghorabaee et al., 2021). Therefore, there is a need to objectively compute weights for criteria that are not influenced by the preferences of the decision makers. The objective weighting method used depends on the original decision matrix. The most common objective weighting methods are the entropy method, standard deviation, and criteria importance through intercriteria correlation. The entropy (Shahnazari et al., 2020) method is not applicable for this study because the decision matrix contains negative values. In this study, we proposed a new method based on the coefficient of variation, rather than standard deviation. However, while writing this paper (i.e. after the research had taken place), we discovered a study (Meng et al., 2020) that proposed the same idea for evaluating the importance of a node in a rail network.

The coefficient of variation method measures the degree of data dispersion, with weight of the criterion being positively correlated with the degree of dispersion. Because a smaller dispersion does not provide a useful input toward the decision, it carries a lower weight.

Below are the steps of the detailed COV approach for PET waste management.

Throughout this section we use the following notation:

$A=\{A_{i},\;i=1,2,\ldots ,m\}$: set of PET waste treatment alternatives.

$C=\left\{C_{j},\;j=1,2,\ldots ,n\right\}$: set of performance evaluation criteria.

$x_{ij}$: performance of alternative *i* against performance criterion *j*

*X*: decision matrix, that is, the performance matrix with dimensions m×n

$$X=\left[\begin{array}{ccc} x_{11}\; & \cdots \; & x_{1n}\\ \vdots \; & \ddots \; & \vdots \\ x_{m1}\; & \cdots \; & x_{mn} \end{array}\right]$$

$\mu_{\left|j\right|}$: absolute value of the mean of performance criterion j

${\mathrm{SD}}_{j}$; standard deviation of performance criteria j

${\mathrm{COV}}_{j}$: coefficient of variation of performance criteria j

$W_{j}$: weight of performance criterion j.

The steps of the COV method were as follows:

Step 1: build the performance evaluation matrix $X_{mn}$.

$$X=\left[\begin{array}{ccc} x_{11}\; & \cdots \; & x_{1n}\\ \vdots \; & \ddots \; & \vdots \\ x_{m1}\; & \cdots \; & x_{mn} \end{array}\right]\;\forall j=1,2,\ldots ,n$$

Step 2: compute the standard deviation ${\mathrm{SD}}_{j}$.

$${\mathrm{SD}}_{j}=\sqrt{\frac{\sum_{i=1}^{m}{\left(x_{ij}-\mu_{\left|j\right|}\right)}^{2}}{\left(m-1\right)}}\;\forall j=1,2,\ldots ,n$$

Step 3: compute the coefficient of variation.

$${\mathrm{COV}}_{j}=\frac{{\mathrm{SD}}_{j}}{\mu_{\left|j\right|}}$$

Step 4: normalize the coefficient of variation ${\mathrm{COV}}_{j}$ to obtain the weights of each criterion.

$$W_{j}=\frac{{\mathrm{COV}}_{j}}{\sum_{j=1}^{n}{\mathrm{COV}}_{j}}$$

## 4 Results and discussion

This section presents and analyzes the results of the proposed methodology for PET waste management.

### 4.1 Case study

Driven by its high population growth and economic development rate, Qatar, which is located in one of the driest regions in the world, is among the top countries in terms of per capita consumption of bottled water (General Secretariat for Development Planning, 2009). As a result, it is estimated that Qatar generated in 2019, a total amount of 33,877 tons of PET bottle waste. Almost all of this waste has been landfilled. The continuous annual growth in the amount of PET bottle waste, coupled with the increasing scarcity of land that can be used for landfill, makes the problem of managing PET bottle waste particularly acute.

Thus, we performed a pairwise comparison to systematically compute the weights related to each criterion. The pairwise comparison was conducted through a survey of policymakers in Qatar, targeting upper and middle management. In total, the survey had 37 responses from participants directly involved with environmental legislation: 19 (51.4%) from the government, 12 (32.4%) from the industry, 5 (13.5%) from academia, and one (2.7%) was consultant working for the Ministry. Of the 37 respondents, 7 (19%) were top-level management, 23 (62.2%) were middle-level management, and 7 (19%) were lower-level management. The percentage of top management responses allows a good perspective on the state's strategy related to waste treatment and the preferences related to the performance criteria.

**Figure 4:**
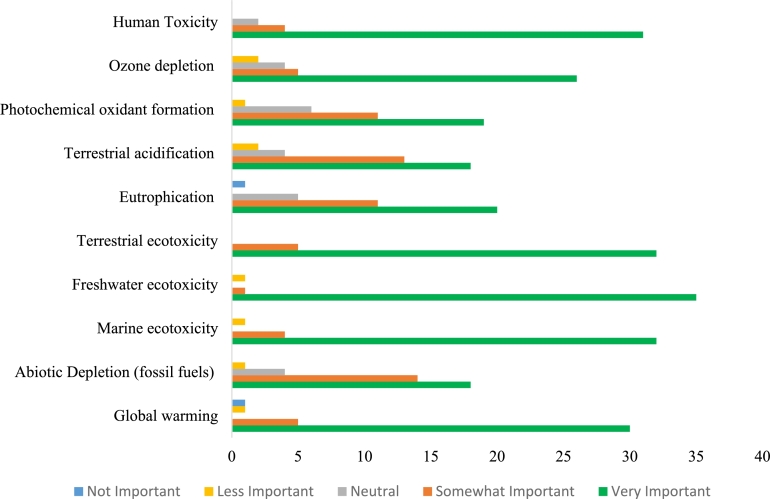
Importance of environmental impact criteria.

Moreover, the analysis of the responses shows that 70% of decision-makers considered environmental performance to be more important than both economic performance and social performance. These results can be explained because 50% of the decision makers were from the Ministry of Environment. The importance of economic and social performances were comparable, with 41% of decision-makers considering social performance to be more important and 59% considering economic performance to be more important. For the sub-economic criteria, 70% of the decision-makers considered cost to be more important than revenue. This result needs to be understood from a Qatar context, since most governmental projects are service projects with no revenue sought from them. Fig. 4 presents the importance of the different sub-environmental performance criteria based on a five-point scale: not important, less important, neutral, somewhat important, and very important. The results of the survey were then used to compute the weights using AHP.

### 4.2 Calculation of the weights

This study considered subjective weights calculated using AHP and objective weights calculated using the COV method. AHP has been used previously to identify the relative weights of a given set of criteria, as indicated in Section 3. However, to the best of our knowledge, the COV method has only been used once with TOPSIS in a study that utilized COV combined with weighted TOPSIS (WTOPSIS) to compute the importance of a node in an urban rail transit (Meng et al., 2020). The COV method eliminates the deviation in the weights calculated using other methods, such as the AHP and Delphi methods, which is caused by subjective factors. The COV method provides objective and accurate weights.

#### 4.2.1 Subjective weights: analytical hierarchy process

AHP calculations are logical and easy to perform. A key step in the approach is to construct the pairwise comparison matrix (Singh, 2021). Recall that if criterion *A* is more important (or as important) as criterion *B*, we need to enter a weight that indicates how many times more important criterion *A* is compared to criterion *B*, and automatically enter its inverse in the transpose position. In this case, the weight must be an integer among {1,...,9}, where a weight of 1 indicates that both criteria are of equal importance, and a weight of 9 means that criterion *A* is extremely more important than criterion *B*.

To mitigate potential biases and thus ensure the reliability of the results, the determination of the pairwise comparison matrix must take into account the opinion of a group of decision makers (obtained through survey results) rather than relying on the opinion of a single decision maker. However, from a practical perspective, it is quite difficult to translate the survey results into a valid pairwise comparison matrix. In this study, we adopt the following approach. First, let us consider two extreme cases. The first is where there are exactly the same number of participants who prefer criterion *A* to criterion *B* and those who think the opposite. In this case, it would be reasonable to consider that the two criteria are of equal importance (i.e. the corresponding weight is 1). In the second case, let us suppose that 100% of the participants prefer criterion *A* to criterion *B*. In this latter case, we can naturally conclude that criterion *A* is extremely more important than criterion *B* (i.e. the corresponding weight is 9). More generally, if the survey results reveal that 100×v% of the participants prefer criterion *A* to criterion *B* (with *v* in [0.5,1]), then the corresponding weight is calculated using the affine transformation in equation (4.1).

(4.1) weight=⌊16v−7⌋,

where ⌊a⌋ is the largest integer that is less than or equal to number *a*. It can be seen that the two extreme cases mentioned above corresponding to v=0.5, and v=1 yield the weights of 1 and 9, respectively.

Using this approach, and based on the survey results that were detailed in Section 4.1, Table 5 presents the obtained pairwise comparison matrix.

**Table 5:** Pairwise comparison matrix of the criteria which hold the preference value.

Criteria	Economic	Environment	Social
Economic	1	0.25	2
Environment	4	1	4
Social	0.50	0.25	1

Subsequently, Table 6 presents the normalized matrix that was obtained by dividing each value by the sum of the entries in the corresponding column. Hence, for Columns 1, 2, and 3, we divided by 6.33, 1.40, and 9, respectively.

**Table 6:** Normalized matrix.

Criteria	Economic	Environment	Social
Economic	0.182	0.17	0.286
Environment	0.727	0.67	0.571
Social	0.091	0.17	0.143

The weights were then calculated by averaging the values of each row (criteria) as follows:

$$W_{1}=\frac{0.182+0.17+0.286}{3}=0.211$$

$$W_{2}=\frac{0.727+0.67+0.571}{3}=0.655$$

$$W_{3}=\frac{0.091+0.17+0.143}{3}=0.133$$

The final weights for the main criteria are presented in Table 7.

**Table 7:** Normalized principal Eigen vector (i.e. weights/priority vector).

Criteria	W_j_
Economic	0.211
Environment	0.655
Social	0.133

After obtaining the weights, the consistency index was calculated using equation (4.2):

(4.2) $$\mathrm{Consistency}\;\mathrm{Index}\;(\mathrm{CI})=\frac{\lambda_{\max}-n}{n-1}$$

*n*: number of the compared criteria

$\lambda_{\max}$: principal eigenvalue.

The $\lambda_{\max}$ was calculated using equation (4.3):

(4.3) $$\mathrm{AW}=\lambda_{\max}W$$

*A*: matrix of pairwise comparison

*W*: computed priority vector

So $\lambda_{\max}=3.054$

$$\mathrm{CI}=\frac{3.054-3}{2}=0.027$$

Finally, the consistency ratio was calculated using equation (4.4):

(4.4) $$\mathrm{CR}=\frac{\mathrm{CI}}{\mathrm{RI}}$$

RI: random consistency index

From (Pachemska et al., 2014) we know that the RI for n=3 is 0.52, so the random consistency index is

$$\mathrm{CR}=\frac{0.027}{0.52}=0.0520$$

The CR was 0.0520, which is below the acceptable inconsistency value of CR≤0.1, indicating that the inconsistency level was acceptable.

The final step was to calculate the final weight for each criterion which presented in Table 8.

**Table 8:** Weights of the criteria and sub-criteria weights calculated using AHP.

Criteria	Sub-Criteria	Weight (W)
Social	Human Toxicity Potential	10.22%

Economic	Cost	14.85%
	Revenue	6.28%

Environment	Global warming	8.22%
	Abiotic Depletion	6.63%
	Marine ecotoxicity	8.60%
	Freshwater ecotoxicity	8.97%
	Terrestrial ecotoxicity	8.70%
	Eutrophication	6.81%
	Terrestrial acidification	6.53%
	Photochemical oxidant formation	6.66%
	Ozone depletion	7.54%

#### 4.2.2 Objective weights: coefficient of variation method

The weights of the different performance criteria were calculated using the COV method. The first step was to determine the standard deviation of each criterion j using the equation (4.5):

(4.5) $${\mathrm{SD}}_{j}=\sqrt{\frac{\sum_{i=1}^{m}{\left(x_{ij}-\bar{X_{j}}\right)}^{2}}{m}}\;\forall j=1,2,\ldots ,n$$

After that, the COV for each criteria j was calculated using the equation (4.6):

(4.6) $${\mathrm{COV}}_{j}=\frac{{\mathrm{SD}}_{j}}{\left|\mu_{j}\right|}$$

Then, the sum of all COV was calculated to be 22.4.

Finally, the weights of each criterion were calculated using the equation (4.7):

(4.7) $$W_{j}=\frac{{\mathrm{COV}}_{j}}{22.4}$$

The results are illustrated in Table 9.

**Table 9:** Calculated weights using the COV method.

Process	COV_j_	W_j_
GWP	1.3	5.82%
ADP	1.557	6.96%
METP	2.0899	9.33%
FETP	1.024	4.57%
TETP	1.13	5.05%
EP	1.358	6.07%
TAP	1.528	6.83%
POFP	3.09	13.81%
ODP	2.092	9.35%
HTP	5.66	25.29%
Cost	0.67	3.08%
Revenue	0.8637	3.85%

### 4.3 TOPSIS

#### 4.3.1 TOPSIS-AHP

This section presents the results of the TOPSIS using the weights obtained from the AHP approach. The highest weights were 14.85% and 10.22%, obtained for cost and human toxicity potential, respectively. The final ranking of the alternatives illustrates that the optimal technique for PET treatment was closed-loop recycling, followed by pyrolysis, open-loop mechanical recycling, and open-loop semi-mechanical recycling as presented in Table 10.

**Table 10:** Measurement of the ideal separation ($S_{i}^{+}$), negative-ideal separation ($S_{i}^{-}$), and relative closeness for TOPSIS-AHP.

Process	$S_{i}^{+}$	$S_{i}^{-}$	$S_{i}^{+}+S_{i}^{-}$	***C***_***i***_	Rank
Closed-loop	3.01 × 10^−2^	2.61 × 10^−1^	2.91 × 10^−1^	0.897	1
Open-loop (mechanical)	1.09 × 10^−1^	1.89 × 10^−1^	2.98 × 10^−1^	0.635	3
Open-loop (semi-mechanical)	1.12 × 10^−1^	1.86 × 10^−1^	2.99 × 10^−1^	0.624	4
Landfill	1.42 × 10^−1^	1.75 × 10^−1^	3.16 × 10^−1^	0.553	6
Incineration with heat recovery	2.01 × 10^−1^	1.33 × 10^−1^	3.34 × 10^−1^	0.397	8
Incineration with heat and power recovery	1.57 × 10^−1^	1.98 × 10^−1^	3.55 × 10^−1^	0.558	5
Glycolysis	1.70 × 10^−1^	1.61 × 10^−1^	3.31 × 10^−1^	0.485	7
Pyrolysis	1.12 × 10^−1^	2.04 × 10^−1^	3.16 × 10^−1^	0.646	2

Based on the TOPSIS-AHP approach, closed-loop recycling was shown to be the optimal treatment for processing PET waste considering different sustainability pillars. To confirm the results, the analysis was repeated using objective weights computed using the COV method. The results are presented in the next section.

#### 4.3.2 TOPSIS-COV

The COV approach measures the degree of dispersion of the data, meaning that the higher the dispersion, the higher the weight of the criterion. The logic behind using this method is that the dispersion of results will have a higher impact on the criteria weight and provide more information about the treatment alternatives. When the dispersion is small, the criterion does not have a significant impact on its rank.

The results of the TOPSIS-COV confirmed the previous results obtained from TOPSIS-AHP, with closed-loop recycling being the top ranked method when considering all performance criteria as shown in Table 11.

**Table 11:** Measurement of the ideal separation ($S_{i}^{+}$), negative-ideal separation ($S_{i}^{-}$), and relative closeness for TOPSIS-COV.

Process	$S_{i}^{+}$	$S_{i}^{-}$	$S_{i}^{+}+S_{i}^{-}$	***C***_***i***_	Rank
Closed-loop	9.0 × 10^−3^	4.10 × 10^−1^	4.19 × 10^−1^	0.979	1
Open-loop (mechanical)	1.94 × 10^−1^	2.56 × 10^−1^	4.50 × 10^−1^	0.569	2
Open-loop (semi-mechanical)	1.97 × 10^−1^	2.53 × 10^−1^	4.50 × 10^−1^	0.563	3
Landfill	2.21 × 10^−1^	2.32 × 10^−1^	4.53 × 10^−1^	0.512	5
Incineration with heat recovery	2.56 × 10^−1^	2.14 × 10^−1^	4.70 × 10^−1^	0.456	7
Incineration with heat and power recovery	3.55 × 10^−1^	1.73 × 10^−1^	5.29 × 10^−1^	0.328	8
Glycolysis	2.10 × 10^−1^	2.48 × 10^−1^	4.57 × 10^−1^	0.542	4
Pyrolysis	2.41 × 10^−1^	2.21 × 10^−1^	4.62 × 10^−1^	0.479	6

### 4.4 Comparison

The analysis included subjective weights and objective weights generated using the AHP and COV methods, respectively. The advantage of the AHP method is that it considers the preferences of the decision makers and can thus theoretically provide an indication of the strategy and direction of the State of Qatar in the areas of waste management and environmental protection, allowing optimal projects aligned with the national strategy to be selected. However, the disadvantage of AHP is that it is influenced by the emotions and previous experiences of the decision-makers, as well as by the number of criteria considered.

**Figure 5:**
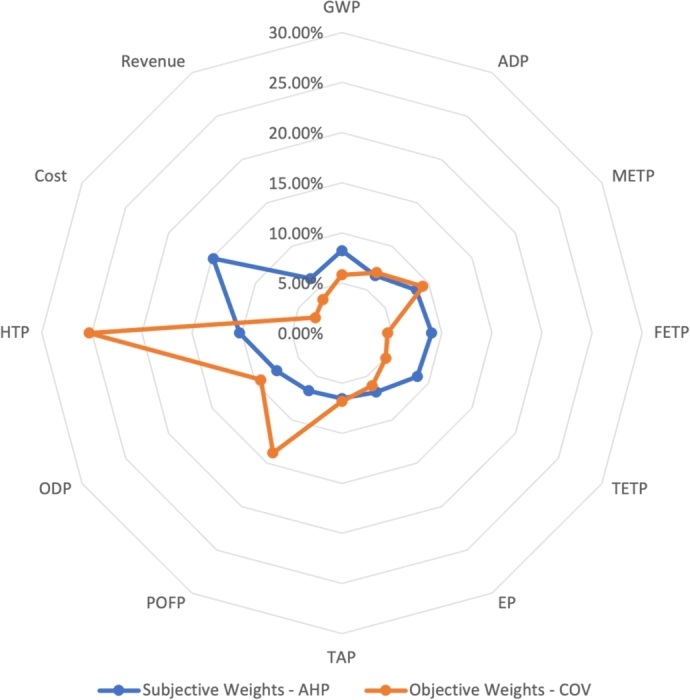
Difference between the rank of the processes using subjective and objective weights. Global Warming Potential (GWP), Abiotic Depletion Potential (ADP), Marine Ecotoxicity Potential (METP), Freshwater Ecotoxicity Potential (FETP), Terrestrial Ecotoxicity Potential (TETP), Eutrophication Potential (EP), Terrestrial Acidification Potential (TAP), Photochemical Oxidant Potential (POFP), Ozone Depletion Potential (ODP), and Human Toxicity Potential (HTP).

As shown in Fig. 5, the highest differences between the weights obtained by the two methods were for human toxicity potential, cost, and photochemical oxidant potential. The weight of cost decreased by 11.77% when calculated using the COV method compared to the AHP method, which can be explained by the small variance in the cost related to each process. The low degree of desperation in the cost data available in the literature and presented in Table 4 is the reason of the reduction on the cost weight. This implies that cost should not significantly impact the final rank of the processes. However, using AHP, cost is an important factor for decision-makers in selecting future ventures, so the weight is higher. On the other hand, the weight of the human toxicity potential increased by 15% when calculated using COV because the differences were quite noticeable between the different processes. Thus, human toxicity potential should have a greater impact on the final rank of the techniques. The weight of freshwater ecotoxicity potential indicator was about 4.4% less using COV. The difference in this potential is because it had lower dispersion in terms of the data, however since Qatar has a problem in the availability of freshwater it has higher importance to the decision-makers. Similarly, photochemical oxidant potential increased by 7.15%. The large dispersion in the value of these performance indicators gave it a higher weight in the COV approach.

Despite these differences, both AHP and COV suggested that closed-loop recycling is the optimal technique for processing PET bottle waste. The results can be explained by the savings offered by closed-loop recycling in terms of the virgin material needed to make the same amount of PET bottles, including all energy, emissions, and other factors. While other processes, such as pyrolysis, open-loop, and heat and energy recovery, offer the creation of new products, it does not impact the need to make new bottles; therefore, the environmental burden will continue to be the same.

### 4.5 Sensitivity analysis

**Figure 6:**
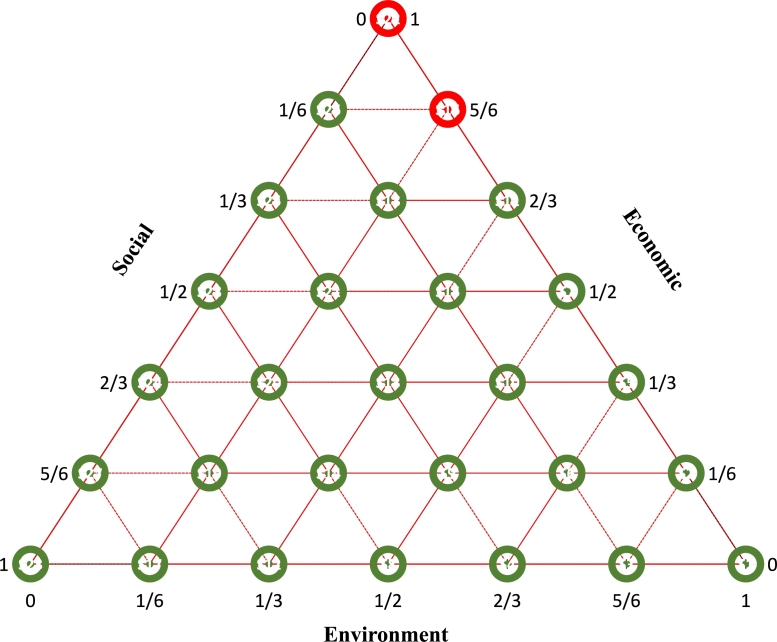
Sensitivity analysis (Green: Closed-Loop Recycling, Red: Pyrolysis).

A sensitivity analysis was performed for the weights of the three main performance indicators to investigate how changes in the weights will impact the optimal technology for the treatment of PET waste (Fig. 6). The weights considered in the analysis for the three main criteria for all scenarios are detailed in Table 12. The sensitivity analysis consisted of 28 scenarios with different weights. Closed-loop recycling was determined to be the optimal technology for PET waste treatment in 26 (92.9%) of scenarios, with pyrolysis considered optimal in the remaining 2 (7.1%).

**Table 12:** List of the weights for the main criteria for each scenario.

Scenario	Environment	Economic	Social
S1	1	0	0
S2	5/6	1/6	0
S3	5/6	0	1/6
S4	2/3	0	1/3
S5	2/3	1/3	0
S6	2/3	1/6	1/6
S7	1/2	1/2	0
S8	1/2	0	1/2
S9	1/2	1/6	1/3
S10	1/2	1/3	1/6
S11	1/3	2/3	0
S12	1/3	0	2/3
S13	1/3	1/3	1/3
S14	1/3	1/6	1/2
S15	1/3	1/2	1/6
S16	1/6	5/6	0
S17	1/6	0	5/6
S18	1/6	2/3	1/6
S19	1/6	1/6	2/3
S20	1/6	1/3	1/2
S21	1/6	0.5	1/3
S22	0	1	0
S23	0	0	1
S24	0	1/3	2/3
S25	0	2/3	1/3
S26	0	5/6	1/6
S27	0	1/6	5/6
S28	0	1/2	1/2

Pyrolysis superseded closed-loop recycling in cases where environmental indicators had either zero weight or a weight of 16.6% with zero weight for the social indicator. The sensitivity analysis robustly proved that closed-loop recycling technology is optimal for treating PET waste, considering different aspects of the environment, economy, and society.

## 5 Conclusions

In this study, eight different PET waste treatment methods were compared and analyzed against twelve different performance criteria representing the pillars of sustainability: environment, economic, and social. This study considered eight technologies that have been well-developed and used for the treatment of PET bottles using environmental performance criteria of global warming potential, abiotic depletion potential, marine ecotoxicity potential, freshwater ecotoxicity potential, terrestrial ecotoxicity potential, eutrophication potential, terrestrial acidification potential, photochemical oxidant formation, and ozone depletion potential; economic criteria of the cost of the technology and revenue; and social criteria of human toxicity potential.

Both approaches determined closed-loop recycling to be the optimal technique for processing PET waste bottles in Qatar. The weights of all performance indicators, except cost, photochemical oxidant, and human toxicity, were comparable when calculated using the two methods. The weight of the cost was increased by approximately 12% using the AHP approach, reflecting the decision makers' preference to reduce costs for any future ventures. The accuracy of this research can be further improved if life cycle analyses were available specifically for the State of Qatar, which represents the current PET waste management system. However, this requires a systematic process of data collection and analysis for all end-of-life processes. In addition, a second avenue of research worth exploring as a natural extension of this study is the optimization of the PET waste collection and closed-loop recycling process.

## CRediT authorship contribution statement

### Author contribution statement

**Nayla Ahmad Al-Thani; Mohamed Haouari:** Conceived and designed the experiments; Performed the experiments; Analyzed and interpreted the data; Contributed reagents, materials, analysis tools or data; Wrote the paper.

**Tareq Al-Ansari:** Conceived and designed the experiments; Contributed reagents, materials, analysis tools or data; Wrote the paper.

### Funding statement

This work was supported by the Awards GSRA4-1-0524-17104 from 10.13039/100008982Qatar National Research Fund (a member of the Qatar Foundation). The contents herein are solely the responsibility of the authors.

### Data availability statement

Data included in article/supplementary material/referenced in article.

### Declaration of interests statement

The authors declare no conflict of interest.

### Additional information

No additional information is available for this paper.
